# Supplementary material for: Increase in antioxidant capacity associated with the successful subclone of hypervirulent carbapenem-resistant Klebsiella pneumoniae ST11-KL64
Source: Nat Commun. 2024 Jan 2;15:67. doi: 10.1038/s41467-023-44351-3 (PMC10761919; doi:10.1038/s41467-023-44351-3)
Supplement: Supplementary file 1 — Supplementary information [file 41467_2023_44351_MOESM1_ESM.pdf]

Increase in antioxidant capacity associated with the successful subclone of hypervirulent carbapenem-resistant *Klebsiella pneumoniae* ST11-KL64
Supplementary information

Supplementary Tables

Supplementary Table 1. The marginal likelihood estimations of different sampling approaches for ST11 chromosomes sequence dataset.

		Strict clock	Relaxed clock
239 samples	No sampling dates	-1067929.759	-1068012.843
	Sampling dates	-1068631.785	-1068428.287
	log(Bayes factor)	-702.0268036	-415.4439623
220 samples	No sampling dates	-1867271.552	-1867413.898
	Sampling dates	-1867265.781	-1867650.18
	log(Bayes factor)	5.77037814	-236.2813913
218 samples	No sampling dates	-1867450.523	-1867442.01
	Sampling dates	-1867346.156	-1867515.105
	log(Bayes factor)	104.3664919	-73.0946159

Supplementary Table 2. Statistical analysis of specific genes from virulence plasmids based on various serotypes.

Serotype	Gene	Description	Sensitivity	Specificity	*p* value*
KL64 (*n* = 65)	*g142*	DsbA family protein	95.24	87.14	2.83E-12
KL1 (*n* = 83)	*g280*	*hp2* in ~3 Kb region	86.84	98.11	2.36E-18
	g417	*hp1* in ~3 Kb region	100	94.34	1.76E-22
	g499	*hp3* in ~3 Kb region	100	94.34	1.76E-22
KL2 (*n* = 42)	*g235*	Hypothetical protein	88.46	92.31	9.70E-14
	g521	Hypothetical protein	96.15	92.31	2.15E-16
	g536	GNAT family N-acetyltransferase	88.46	92.31	9.70E-14
	ymoA	Global Modulating protein	96.15	87.69	1.99E-14

*Sensitivity, the sensitivity if using the presence of this gene as a diagnostic test to determine trait-positivity. Specificity, the specificity if using the non-presence of this gene as a diagnostic test to determine trait-negativity. The *p* value, the naïve *p*-value for the null hypothesis that the presence/absence of this gene is unrelated to the trait status by two-sided Fisher's exact test. Serotypes were used as traits. The result was obtained using Scoary v1.6.16[1]. Source data are provided as a Source Data file.

Supplementary Table 3. The representative strains used for the function study of deletion region of virulence plasmids.

Strain No.	Similarity to pK2044	Collection date	Specimen type	Serotype	MLST	Virulence plasmid type	KPC plasmid type
C4599	99%	2017-9-12	sputum	KL1	ST23	IncHI1B(pNDM-MAR)/repB	IncFII(pHN7A8)/IncR
C1789	95%	2016-8-1	urine	KL64	ST11	IncHI1B(pNDM-MAR)/repB	IncFII(pHN7A8)/IncR

Supplementary Table 4. Definition of four categories of *K. pneumoniae* in the study.

	Resistance score*	Virulence score*
Classic *K. pneumoniae* (cKP)	< 2	≤ 2
Hypervirulent *K. pneumoniae* (hvKP)	< 2	> 2
Carbapenem resistant *K. pneumoniae* (CRKP)	≥ 2	≤ 2
Hypervirulent CRKP (hv-CRKP)	≥ 2	> 2

*What the scores represent can be found here:
https://github.com/katholt/Kleborate/wiki/Scores-and-counts

Supplementary Table 5. The plasmid vectors and strains for genome editing assays used in the study.

Strains or plasmids	Description
C1789::pEASY	A KPC-2-carrying ST11 hv-CRKP with a p*EASY*-T1 vector
C1789::hp1-4	C1789 with a p*EASY*-T1 vector reversed *hp1-4* genes
C1789::hp5	C1789 with a p*EASY*-T1 vector reversed *hp5* gene
C4599Δ3K	A 3 Kb region knock-out ST23 CR-hvKP C4599
C4599Δ3K::pEASY	C4599Δ3K with a p*EASY*-T1 vector
C4599Δ3K::hp1-4	C4599Δ3K with a p*EASY*-T1 vector reversed *hp1-4* genes
C4599Δ3K::hp5	C4599Δ3K with a p*EASY*-T1 vector reversed *hp5* gene
p*EASY*-T1	A vector with tigecycline resistance used for gene reversed
pSGKP	A vector with tigecycline resistance used for knockout
pCasKP	A vector with apramycin resistance used for knockout

Supplementary Figures

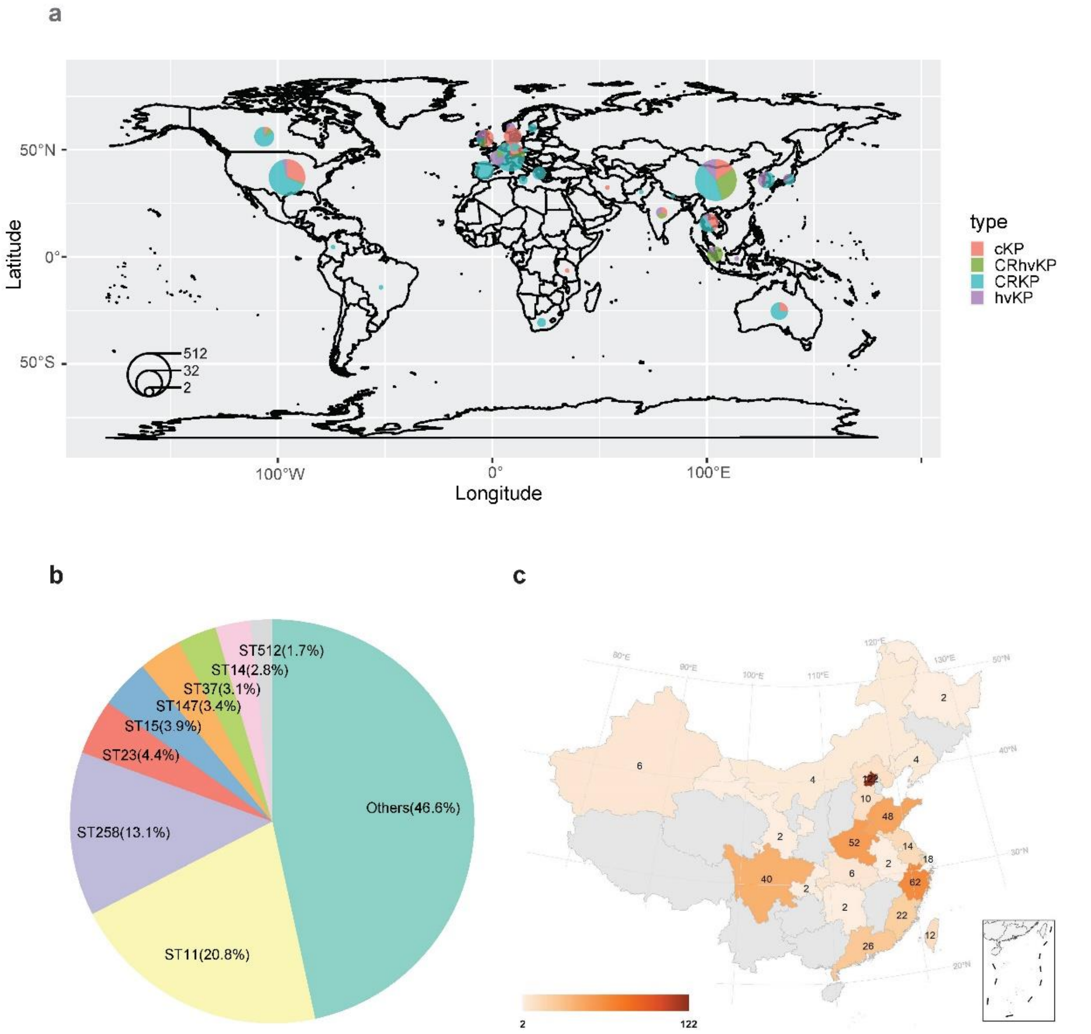

Supplementary Figure 1. The distribution of *K. pneumoniae* genome assemblies in the study. Global distribution of *K. pneumoniae* genome assemblies with geographic information (n = 774) across 31 different countries in the study (**a**). Distribution of MLST in 1,219 *K. pneumoniae* (**b**). Distribution of ST11 *K. pneumoniae* collected from China (n = 228) (**c**). The maps here are schematic. The packages ggplot2[2] and drawMap (https://github.com/psychbruce/drawMap) were used for visualization in R v4.3.1 (https://www.r-project.org/). Source data are provided as a Source Data file.

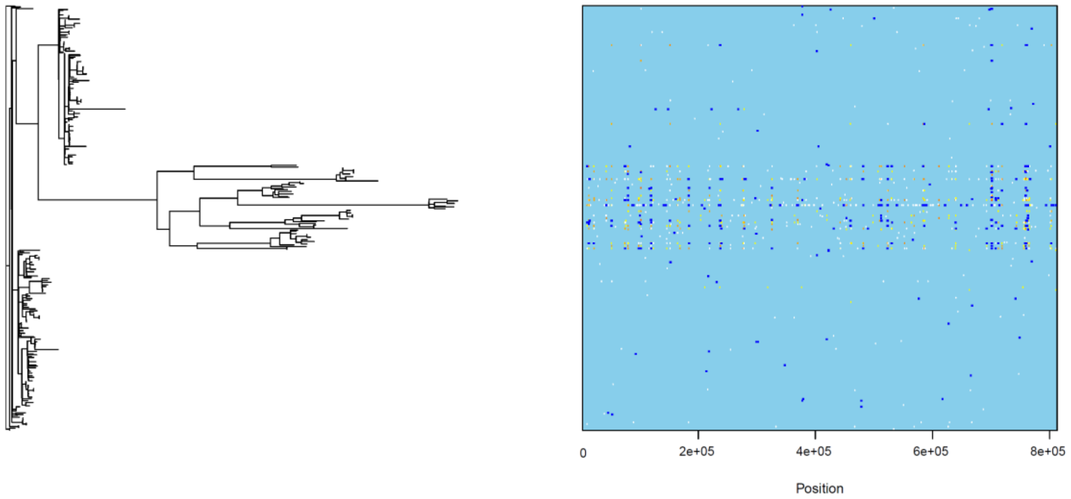

Supplementary Figure 2. The recombination region of ST11 chromosome. Based on the alignment of 246 sequences with 813,526 bp, a recombination filtered multiple sequence alignment with a length of 758,264 bp was constructed. The results of recombination regions were visualized using the recommended Rscript cfml_results.R provided by the clonalframeML[3]. Source data are provided as a Source Data file.

a

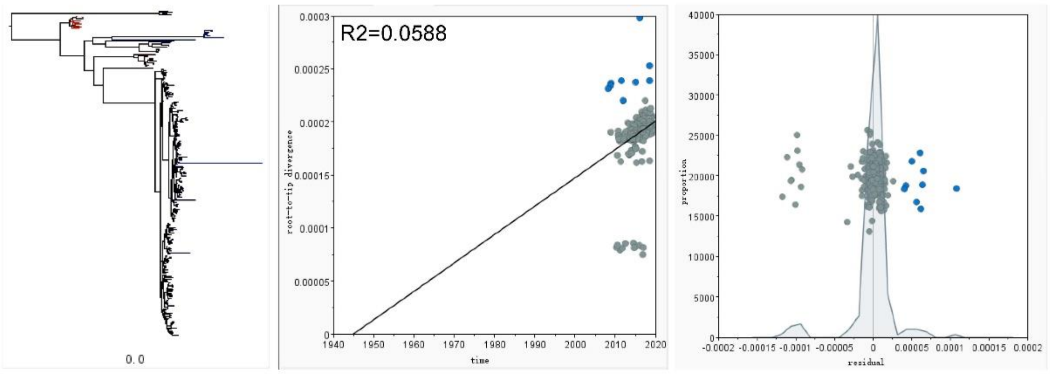

b

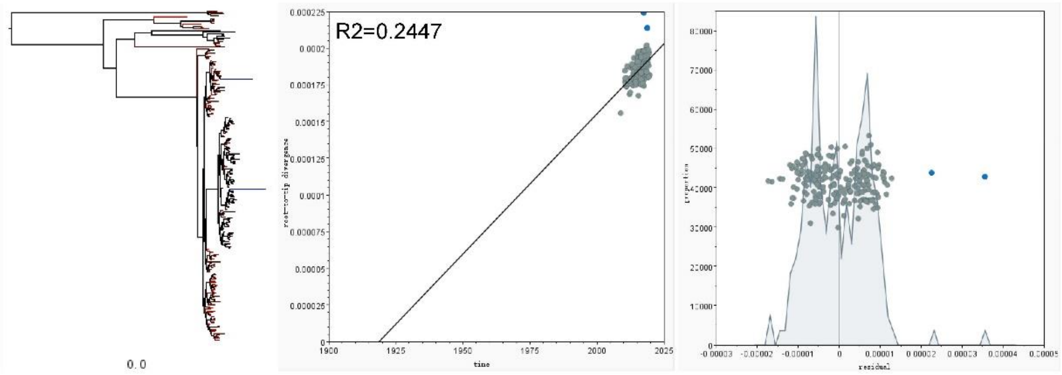

c

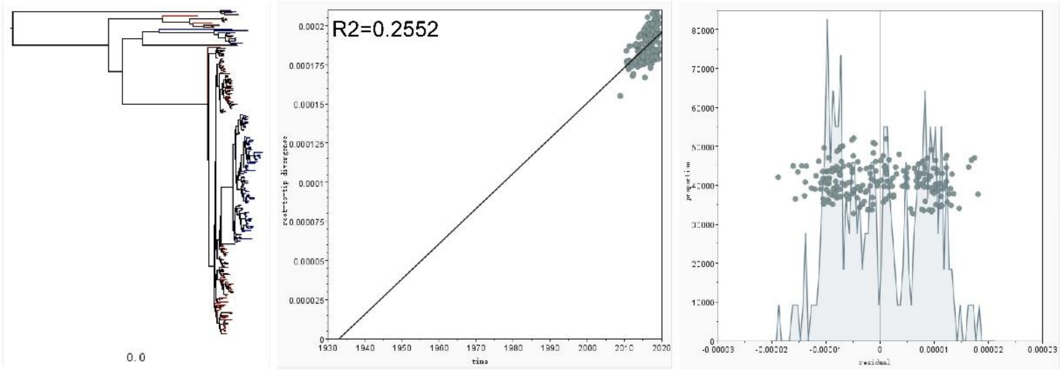

Supplementary Figure 3. Temporal signal of ST11 *Klebsiella pneumoniae*. The dataset comprised a total of 239 samples with collection date (**a**). After removing outliers, the dataset consisted of 220 samples with collection date (**b**). Further removal of outliers resulted in a subset of 218 samples with collection date (**c**). Data visualize using TempEst[4].

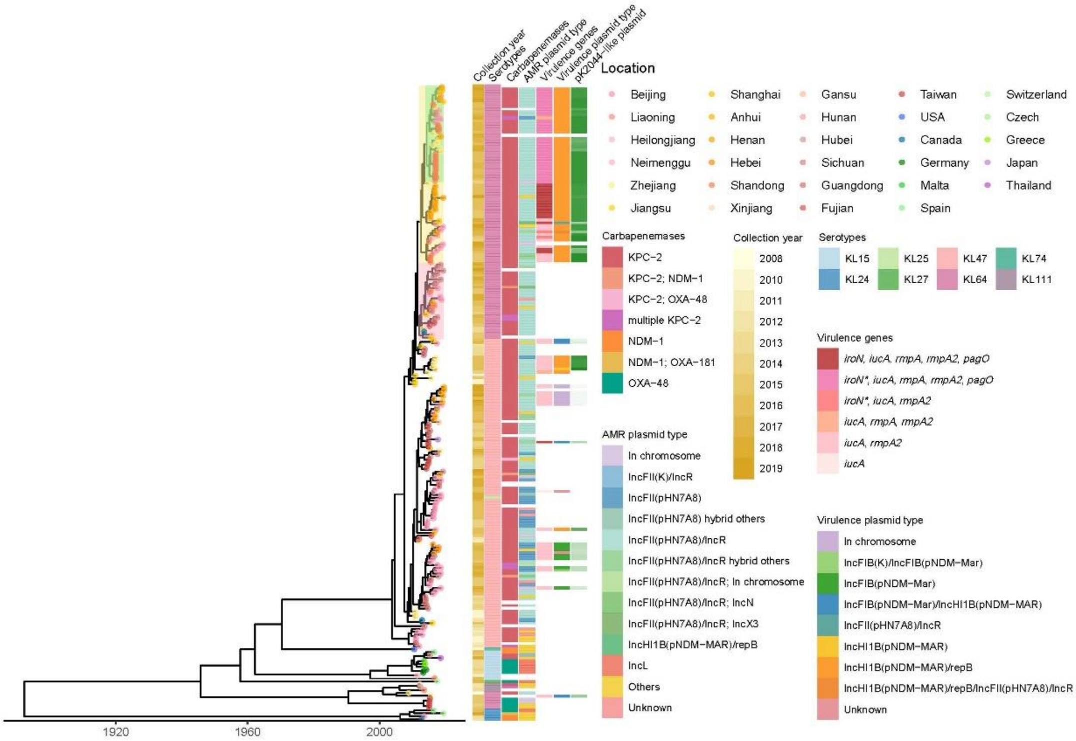

Supplementary Figure 4. A dated phylogenetic tree of ST11 CRKP chromosome. Tip points of the phylogenetic tree were dotted by location where strains were isolated. Heatmap denoted (from left to right) collection date, serotypes, the presence of carbapenemase genes, types of carbapenem resistance plasmids, the presence of virulence genes, types of virulence plasmids and the similarity to pK2044. *iroN** is an incomplete gene, with only the first 1,306 bp mapping out of a total length of 2,181 bp. The similarity to pK2044 was measured by the coverage of comparison of pK2044 and each plasmid using BLASTn[5]. Source data are provided as a Source Data file.

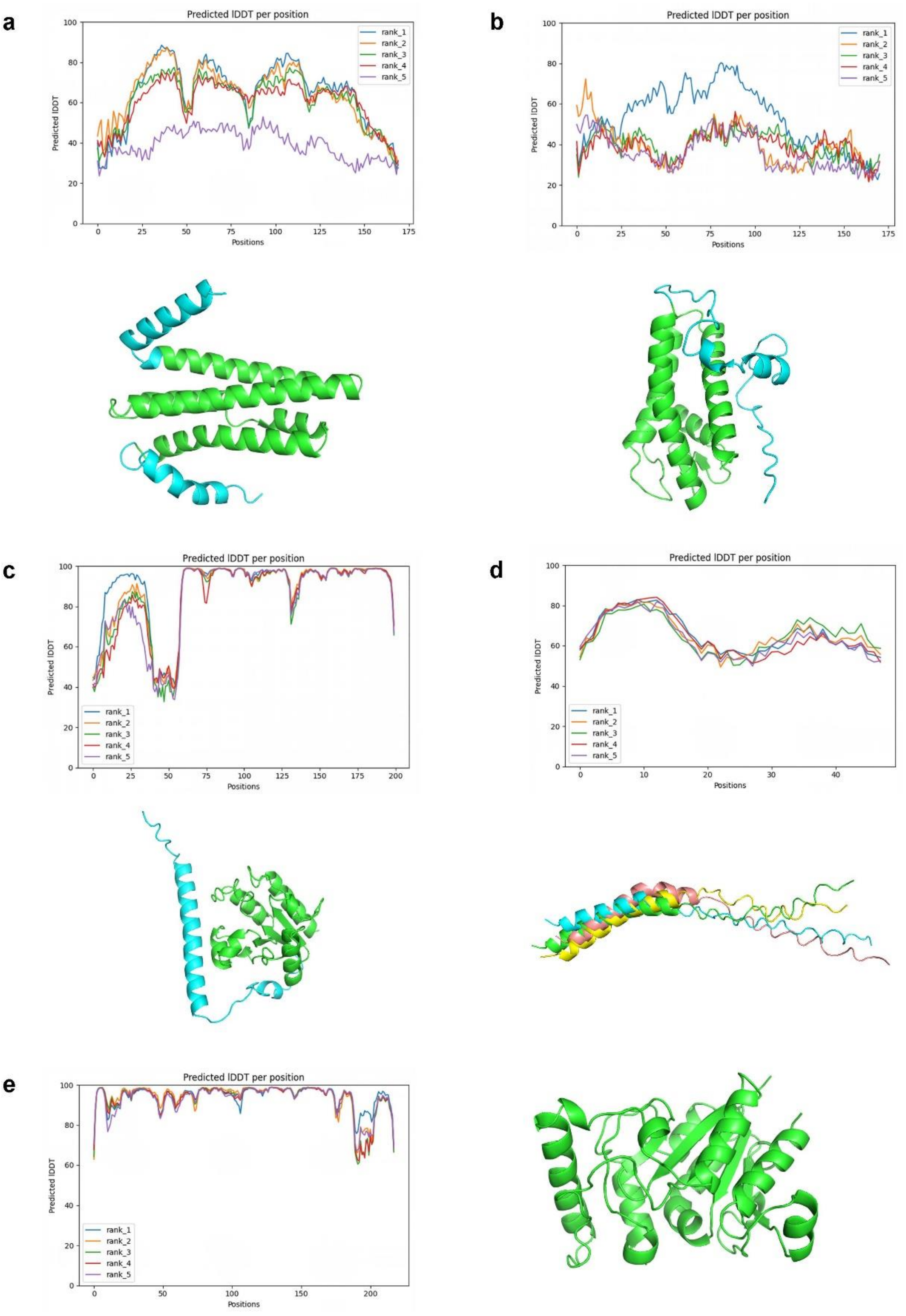

Supplementary Figure 5. Prediction of structure model of hypothetical proteins Hp1-5. The predicted local Distance Difference Test (pLDDT) in the left was a superposition-free score which evaluates local distance differences in a model compared to a reference structure. The right showed prediction of Hp1 (**a**), Hp2 (**b**), Hp3 (**c**), Hp4 (**d**) and Hp5 (**e**) structure model. The N terminus (blue) with low confidence was deleted in analysis, and the green part was searched in the AlphaFold Database (https://alphafold.com). Source data are provided as a Source Data file.

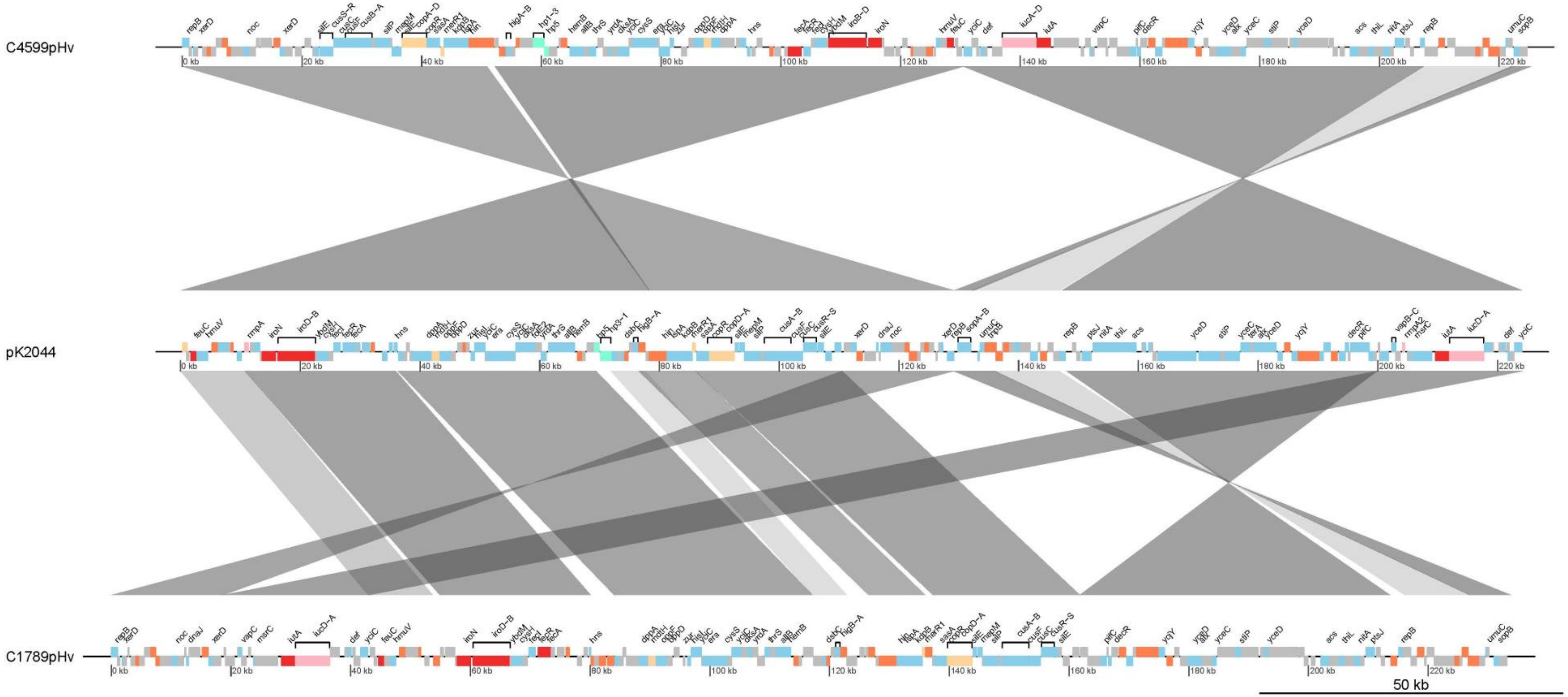

Supplementary Figure 6. The comparison of pK2044 and virulence plasmids on representative strains. Orange blocks: mobile elements; green blocks: *hp1-3* and *hp5* in ~3K region; grey blocks: hypothetical genes; red or pink blocks: virulence genes; yellow blocks: Cu-related genes; blue blocks: genes with names or known products. C1789: ST11-KL64 hv-CRKP; C4599: ST23-KL1 CR-hvKP. Source data are provided as a Source Data file.

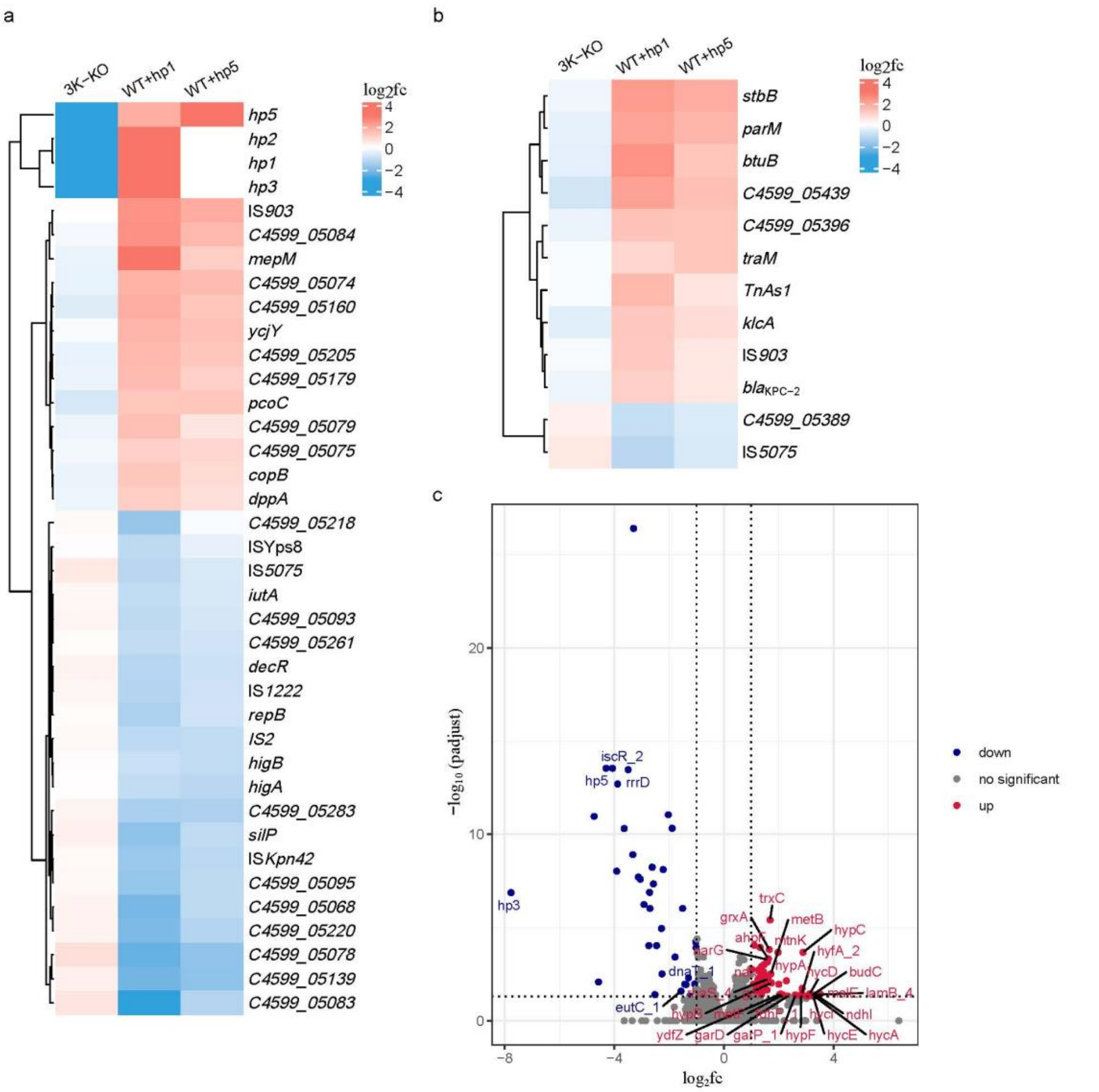

Supplementary Figure 7. Differentially expressed genes of plasmids and total mRNA. Heatmap of DEGs on the virulence plasmid (**a**) and the KPC plasmid (**b**) between C4599 *vs.* C4599Δ3K (3K-KO), C1789::pEasy *vs.* C1789::*hp1-4* (WT+hp1), and C1789::pEasy *vs.* C1789::*hp5* (WT+hp5). Red stripes in the figure represented high expression genes, while blue stripes represented low expression genes. Only significantly changed genes in any of the three groups were shown in each graph (a) and (b). Volcano map of DEGs between C4599 and C4599Δ3K (**c**). The *x*-axis was the $\log_2$ scale of the fold change of gene expression ($\log_2$fc). Negative values indicated downregulation; positive values indicated upregulation. The *y*-axis was the minus $\log_{10}$ scale of the adjusted *p* values [$-\log_{10}$(padjust)], which indicate the significant level of expression difference. The red dots represented significantly upregulated genes with at least two-fold change, while the blue dots represented significantly downregulated genes with at least a half change. Gene names were marked next to the dots if they had. The data was visualized using ComplexHeatmap v2.16.0[6] and ggplot2 v3.4.4[2] in R. Source data are provided as a Source Data file.

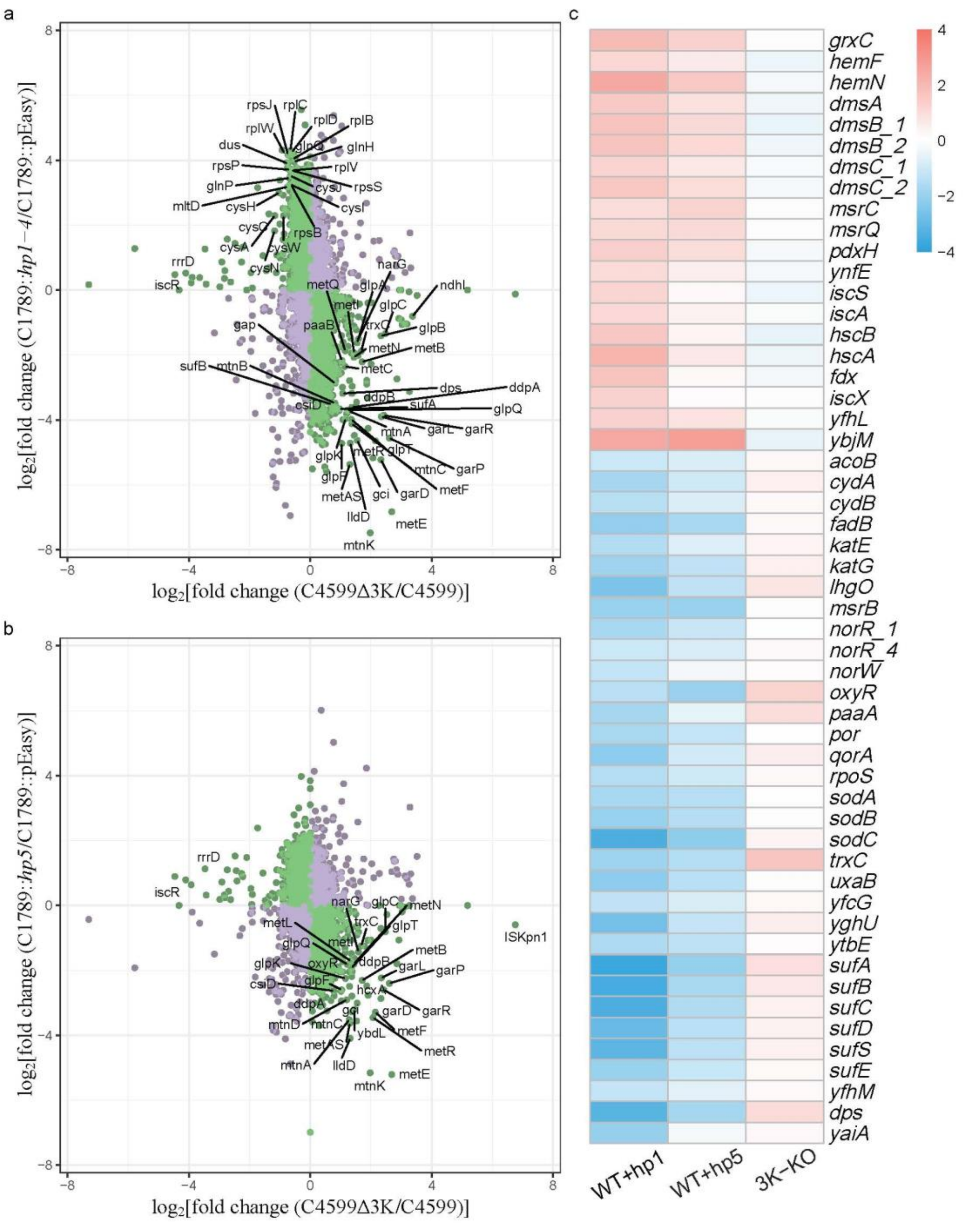

Supplementary Figure 8. Differentially expressed genes of total mRNA between wildtype *vs.* the knockout and wildtype vs. the reversed strains. Scatter diagrams of DEGs between the knock-out and reversed strains (**a**) and (**b**). The *x*-axis was the $\log_2$ scale of the fold change of gene expression between C4599 and C4599Δ3K. The *y*-axis was the $\log_2$ scale of the fold change of gene expression between C1789::pEasy and C1789::*hp1-4* or C1789::*hp5*. Negative values indicated downregulation; positive values indicated upregulation. Compared to the wildtype, genes with opposite changes of the knock-out and the reversed are labeled in green. Some gene names were marked next to the dots if they had. Heatmap of DEGs (**c**) response to oxidative stress between C4599 vs. C4599Δ3K (3K-KO), C1789::pEasy vs. C1789::*hp1-4* (WT+hp1), and C1789::pEasy vs. C1789::*hp5* (WT+hp5). Only DEGs of WT+hp1 as well as genes with WT+hp1/WT+hp5 that have an opposite change trend to 3K-KO are displayed. Red stripes in the figure represented high expression genes, while blue stripes represented low expression genes. The depth of color represents the magnitude of the $\log_2$ scale of the fold change of gene expression. Source data are provided as a Source Data file.

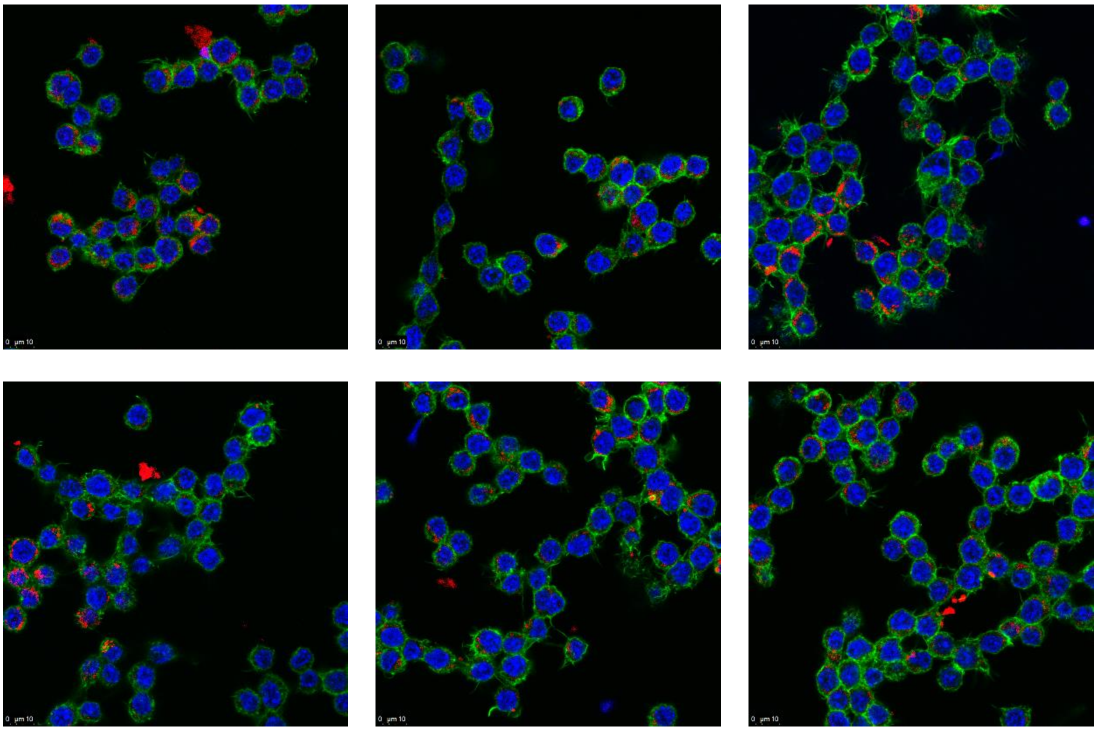

C1789

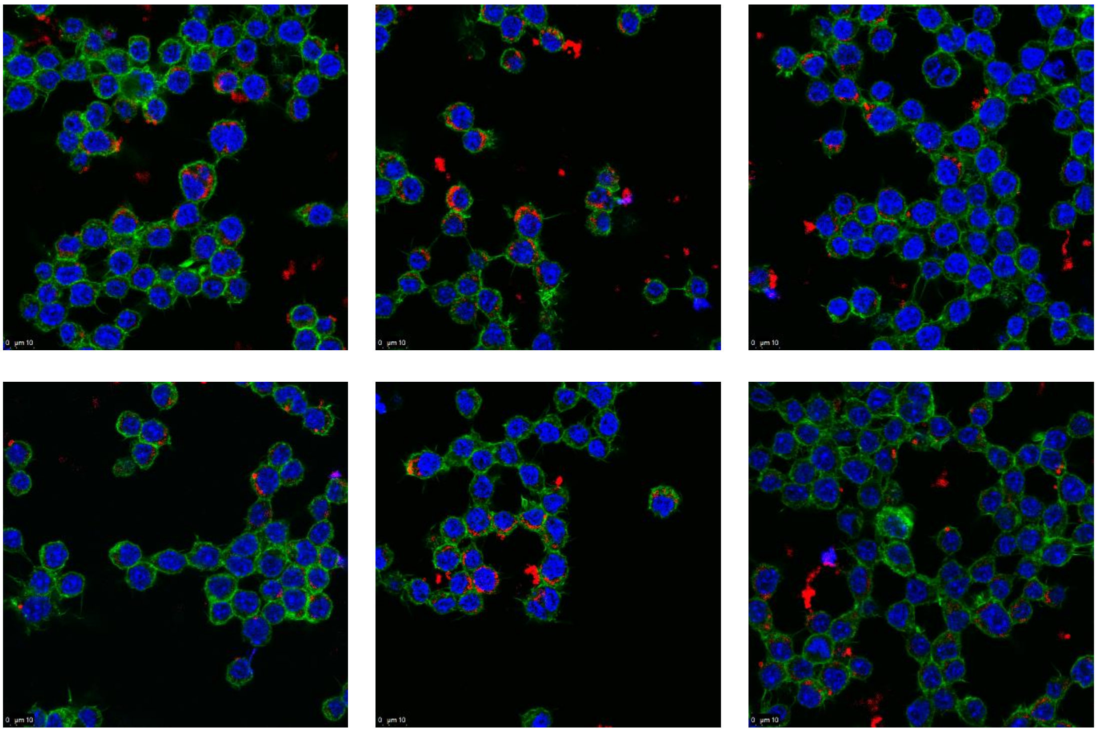

C1789::pEasy

Supplementary Figure 9. Survival in macrophage assay of wildtype and empty vector ST11 hv-CRKP. Utilizing laser confocal microscopy to visualize the survival of bacteria within macrophages. F-actin were stained by rhodamine phalloidin (green), and nuclei were counterstained with DAPI (blue). The bacteria were labeled with pHrodo (red). Scale bar = 10 μm.

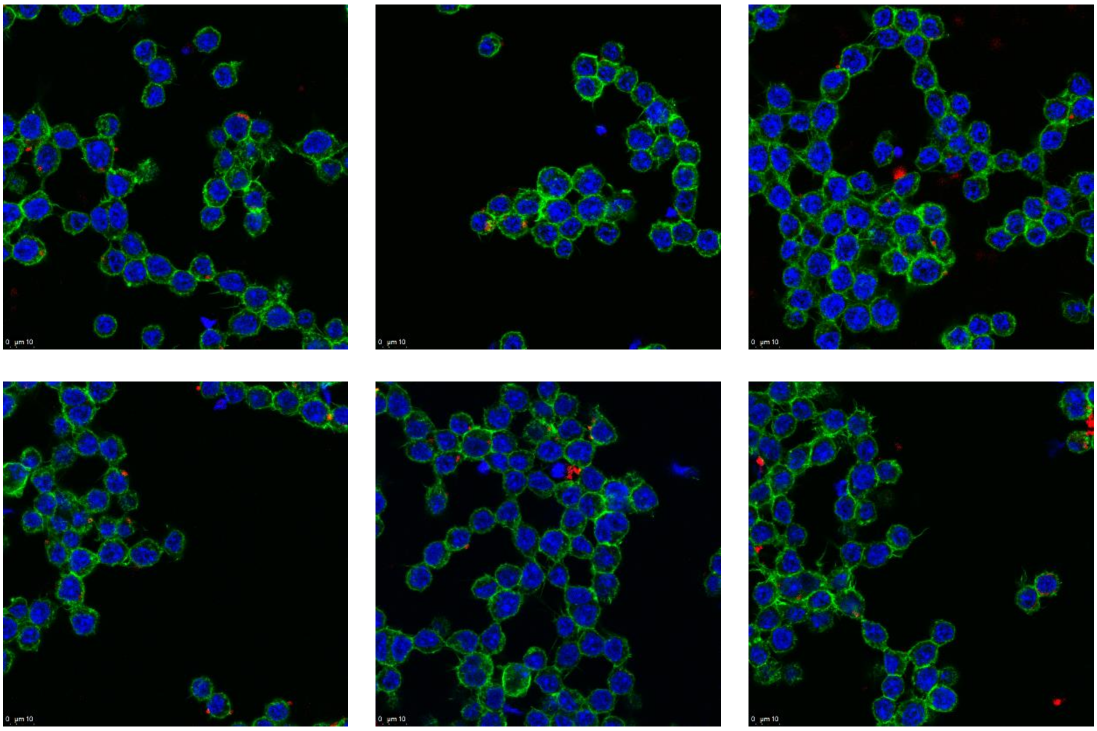

C1789::hp1-4

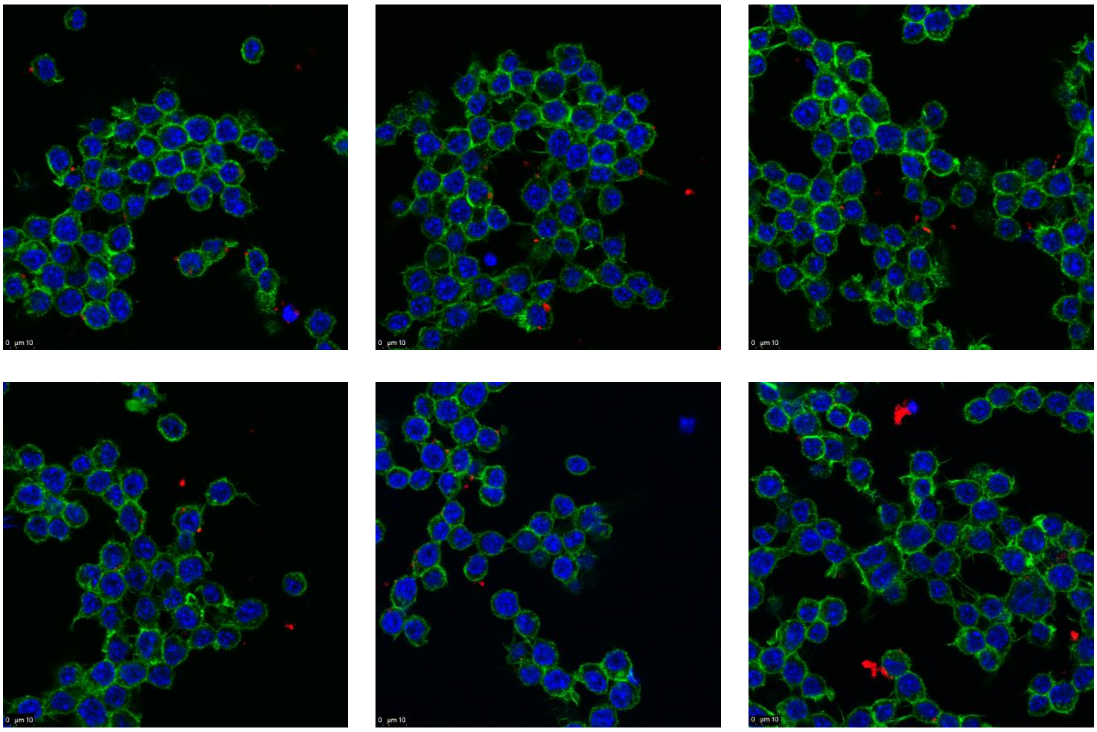

C1789::hp5

Supplementary Figure 10. Survival in macrophage assay of ~3K region reversed ST11 hv-CRKP. Utilizing laser confocal microscopy to visualize the survival of bacteria within macrophages. F-actin were stained by rhodamine phalloidin (green), and nuclei were counterstained with DAPI (blue). The bacteria were labeled with pHrodo (red). Scale bar = 10 μm.

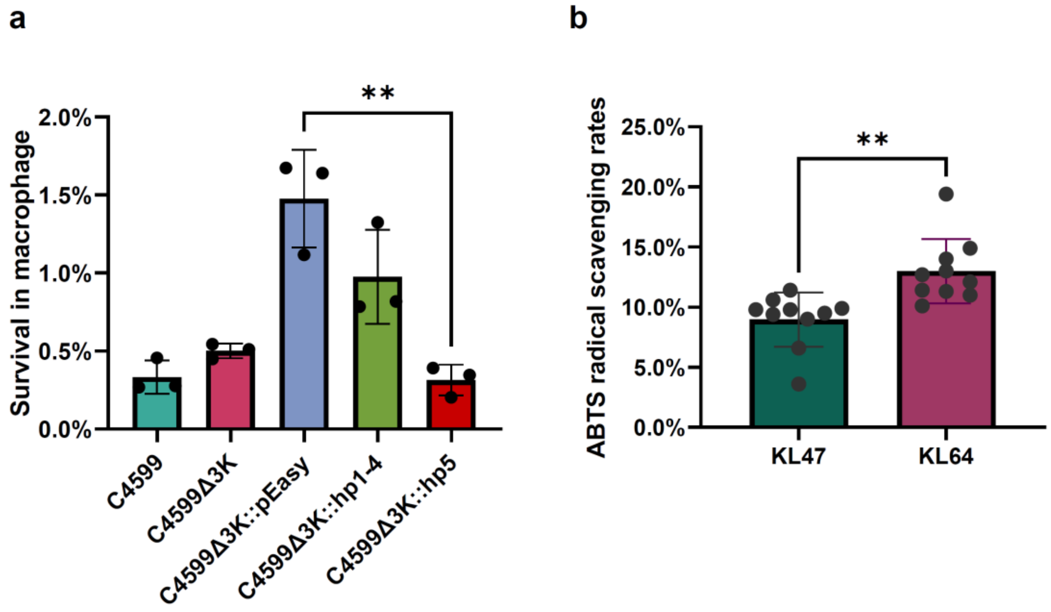

Supplementary Figure 11. Antioxidant capacity of wildtype, ~3K region knockout and reversed ST23 CR-hvKP, ST11-KL47 and ST11-KL64 hv-CRKP. The survival rates of C4599, ~3K knockout and reversed strains in macrophage (**a**). Each strain performed three biological replicates. Two-tailed t-tests were used to compare C4599 vs. C4599Δ3K, C4599Δ3K::pEasy vs. C4599Δ3K::*hp1-4*/C4599Δ3K::*hp5*. ***p* < 0.01 (*p* = 0.004). ABTS activities determination assays of ST11-KL47 and ST11-KL64 hv-CRKP (**b**). Each group comprised ten strains, and each strain performed three technical replicates. The bars and error bars present mean with SD. Two-tailed t-test was evaluated and *p*-values < 0.05 were considered statistically significant. ***p* < 0.01 (*p* = 0.002). Source data are provided as a Source Data file.

References

1. Brynildsrud, O., Bohlin, J., Scheffer, L. & Eldholm, V. Rapid scoring of genes in microbial pan-genome-wide association studies with Scoary. *Genome Biol* **17**, 238 (2016).
2. Wickham, H. *ggplot2: Elegant Graphics for Data Analysis*. (Springer-Verlag New York, 2016).
3. Didelot, X. & Wilson, D. J. ClonalFrameML: efficient inference of recombination in whole bacterial genomes. *PLoS Comput Biol* **11**, e1004041 (2015).
4. Rambaut, A., Lam, T. T., Max Carvalho, L. & Pybus, O. G. Exploring the temporal structure of heterochronous sequences using TempEst (formerly Path-O-Gen). *Virus Evol* **2**, vew007 (2016).
5. Camacho, C. *et al.* BLAST+: architecture and applications. *BMC Bioinformatics* **10**, 421 (2009).
6. Gu, Z. Complex heatmap visualization. *iMeta* **1**, e43 (2022).
