# Supplementary material for: Increase in antioxidant capacity associated with the successful subclone of hypervirulent carbapenem-resistant Klebsiella pneumoniae ST11-KL64
Source: Nat Commun. 2024 Jan 2;15:67. doi: 10.1038/s41467-023-44351-3 (PMC10761919; doi:10.1038/s41467-023-44351-3)
Supplement: Supplementary file 3 — Reporting Summary [file 41467_2023_44351_MOESM3_ESM.pdf]

nature portfolio

Corresponding author(s): Hui Wang
Last updated by author(s): Dec 2, 2023

Reporting Summary

Nature Portfolio wishes to improve the reproducibility of the work that we publish. This form provides structure for consistency and transparency in reporting. For further information on Nature Portfolio policies, see our Editorial Policies and the Editorial Policy Checklist.

Statistics

For all statistical analyses, confirm that the following items are present in the figure legend, table legend, main text, or Methods section.

n/a	Confirmed	
☐	☒	The exact sample size (*n*) for each experimental group/condition, given as a discrete number and unit of measurement
☐	☒	A statement on whether measurements were taken from distinct samples or whether the same sample was measured repeatedly
☐	☒	The statistical test(s) used AND whether they are one- or two-sided<br>*Only common tests should be described solely by name; describe more complex techniques in the Methods section.*
☒	☐	A description of all covariates tested
☐	☒	A description of any assumptions or corrections, such as tests of normality and adjustment for multiple comparisons
☐	☒	A full description of the statistical parameters including central tendency (e.g. means) or other basic estimates (e.g. regression coefficient) AND variation (e.g. standard deviation) or associated estimates of uncertainty (e.g. confidence intervals)
☐	☒	For null hypothesis testing, the test statistic (e.g. *F*, *t*, *r*) with confidence intervals, effect sizes, degrees of freedom and *P* value noted<br>*Give P values as exact values whenever suitable.*
☐	☒	For Bayesian analysis, information on the choice of priors and Markov chain Monte Carlo settings
☒	☐	For hierarchical and complex designs, identification of the appropriate level for tests and full reporting of outcomes
☒	☐	Estimates of effect sizes (e.g. Cohen's *d*, Pearson's *r*), indicating how they were calculated

Our web collection on statistics for biologists contains articles on many of the points above.

Software and code

Policy information about availability of computer code

Data collection	No software was used.
Data analysis	Unicycler v0.4.7, Prokka v1.13.7, Kleborate v0.3.0, Kaptive v2.0.0, Phyre2 v2.0, ColabFold v1.3.0, Foldseek (search.foldseek.com), BLASTn v2.9.0+, PlasmidFinder v2.1, RaxML v8.2.12, Roary v3.13.0, ClonalframeML v1.11, TempEst v1.5.3, SNP-sites 2.5.1, BEAST2 v2.7.4, fastANI v1.33, Snippy v4.6.0, Scoary v1.6.16, Trimmomatic v0.35, fastp v0.20.1, SeqPrep v1.2, Sickle v1.33, Bowtie 2 v2.5.2, RSEM v1.3.3, DESeq2 v3.18, Goatools v1.3.11, KOBAS 2.0, TreeAnnotator v2.7.4, ggtree v3.8.2, genoPlotR v0.8.11, ComplexHeatmap v2.16.0, pheatmap v1.0.12, ggplot2 v3.4.4, drawMap v0.2.0 were used for analysis based on guidelines provided. No custom code or mathematical algorithm was utilized in this study.

For manuscripts utilizing custom algorithms or software that are central to the research but not yet described in published literature, software must be made available to editors and reviewers. We strongly encourage code deposition in a community repository (e.g. GitHub). See the Nature Portfolio guidelines for submitting code & software for further information.

Data

Policy information about availability of data

All manuscripts must include a data availability statement. This statement should provide the following information, where applicable:

- Accession codes, unique identifiers, or web links for publicly available datasets
- A description of any restrictions on data availability
- For clinical datasets or third party data, please ensure that the statement adheres to our policy

KEGG (https://www.kegg.jp/) and GO (https://www.geneontology.org/) database are used in this study. All new assembly data used for phylogenetic analysis have been submitted to in GenBank and assigned the BioProject accession number PRJNA1015184 (https://www.ncbi.nlm.nih.gov/bioproject/PRJNA1015184/). Accession numbers and the processed data generated in this study are provided in the Source Data. Some new assembly data of the total genome dataset (Supplementary Figure 1 showed) are not publicly available for other unfinished projects but are available from the corresponding author on reasonable request. The metadata also can be found in Source Data. Transcriptome data of knockout and reversed strains has been submitted to the Sequence Read Archive (SRA) and assigned the BioProject accession number PRJNA1047885 (https://www.ncbi.nlm.nih.gov/bioproject/PRJNA1047885/).

Research involving human participants, their data, or biological material

Policy information about studies with human participants or human data. See also policy information about sex, gender (identity/presentation), and sexual orientation and race, ethnicity and racism.

Reporting on sex and gender	NA
Reporting on race, ethnicity, or other socially relevant groupings	NA
Population characteristics	NA
Recruitment	NA
Ethics oversight	The Peking University People's Hospital Institutional Review Board (No. 2019PHB194-01) approved this study.

Note that full information on the approval of the study protocol must also be provided in the manuscript.

Field-specific reporting

Please select the one below that is the best fit for your research. If you are not sure, read the appropriate sections before making your selection.

☐ Life sciences ☐ Behavioural & social sciences ☒ Ecological, evolutionary & environmental sciences

For a reference copy of the document with all sections, see nature.com/documents/nr-reporting-summary-flat.pdf

Ecological, evolutionary & environmental sciences study design

All studies must disclose on these points even when the disclosure is negative.

Study description	This study focuses on the genome evolution of ST11 Klebsiella pneumoniae, a major clinical epidemic pathogen, and tries to explore the source and impact of virulence plasmids of newly emergent hypervirulent carbapenem-resistant K. pneumoniae in recent years.
Research sample	A total of 9,930 complete genome data of Klebsiella pneumoniae that the researchers could obtain prior to June 2020 from the NCBI Reference Sequence database (n = 9,093) and our research team own genome database (n = 637).
Sampling strategy	To clarify the chromosome and plasmid, criteria were devised to select publicly available assemblies long enough to be studied, including 1) longest contig length > 4.5 Mb, and 2) number of contigs < 40.
Data collection	The strains collected in this study were derived from several long-term Chinese multicenter surveillances conducted by our research team in the past years, as well as strains collected in previous studies. Most of isolates were from the CRE-network and Chinese Antimicrobial Resistance Surveillance of Nosocomial Infections (CARES) project, which collected strains from hospitals in various provinces and cities in China by local staffs, and were sent to the central laboratory for antimicrobial susceptibility testing and sequencing of resistant or virulent strains. The sampled methods have been described in the previous related articles. Carbapenem-resistant, tigecycline-resistant, colistin-resistant and hypervirulent strains were collected for sequencing. Based on these efforts, a personal sequencing database containing 637 K. pneumoniae has been established. The public genome was directly downloaded from the RefSeq by authors. Then, we generated the genomes togather for the further study. Following filtering according to the criteria above, a total of 1,219 assemblies were considered from the NCBI Reference Sequence database (n = 917) and our database (n = 302).

Timing and spatial scale	The isolates in our own database span the years 2005 to 2019. The data of K. pneumoniae from the RefSeq database prior to June 2020. The strains in the entire dataset were isolated between 2002 and 2019.
Data exclusions	Sequences without collection dates were excluded in the dated phylogenetic analysis.
Reproducibility	All sequences used for analysis have been published in NCBI. The software used for analysis is open source. Most of analyses use the default parameters recommended. Special settings have been mentioned in the article. All attempts at replication were successful.
Randomization	Randomization is not available in this study, because we select the data with specific characteristics from the whole dataset as the object for study. We choose ST11 K. pneumoniae assemblies from database to conduct for phylogenetic analysis after removing a length of longest contig < 4.5 Mb and > 40 total contigs in the assembly. As for plasmid, we focused on plasmids with high similarity (an ANI > 95 and coverage of > 80%) to the classic virulence plasmid pK2044 (NC_006625.1), because high diversity challenged the creation of a sufficiently long multi-sequence alignment to include all isolates.
Blinding	Blinding was not relevant because at that point in time, we collected as much as possible the genome data of all the fully assembled K. pneumoniae that were publicly available around the world and that we could obtain. Sources of bias avoided by blinding did not play a role in this study because, for example, the results of genome analysis are not transferable by human will.

Did the study involve field work? ☐ Yes ☒ No

Reporting for specific materials, systems and methods

We require information from authors about some types of materials, experimental systems and methods used in many studies. Here, indicate whether each material, system or method listed is relevant to your study. If you are not sure if a list item applies to your research, read the appropriate section before selecting a response.

Materials & experimental systems

n/a	Involved in the study
☒	☐ Antibodies
☐	☒ Eukaryotic cell lines
☒	☐ Palaeontology and archaeology
☒	☐ Animals and other organisms
☒	☐ Clinical data
☒	☐ Dual use research of concern
☒	☐ Plants

Methods

n/a	Involved in the study
☒	☐ ChIP-seq
☒	☐ Flow cytometry
☒	☐ MRI-based neuroimaging

Eukaryotic cell lines

Policy information about cell lines and Sex and Gender in Research

Cell line source(s)	RAW264.7, a murine monocytic leukemia cell line, is commonly utilized in scientific experiments. It originates from tumors induced by the male Abelson murine leukemia virus. The cell line was obtained from the Cell Resource Center, Peking Union Medical College (PCRC).
Authentication	None of the cell lines used were authenticated.
Mycoplasma contamination	The cell lines were not tested for mycoplasma contamination recently. The most recent authentication was conducted on March 26, 2019.
Commonly misidentified lines (See ICLAC register)	The cell line is not in the misidentified cell list.
